# A Literary Bombshell

**Published:** 1894-03

**Authors:** 


### A LITERARY BOMB-SHELL.

The Howard Publishing Company, of Detroit and Chicago, have issued part first of "Bacon's Cipher Story," and a copy of it is before us.

Dr. Orville W. Owen, of Detroit, claims to have discovered the cipher, which for many years has been believed to lie hidden in Bacon's works, and by means of it, to have deciphered a "letter to the decipherer." In this letter he found minute instructions how to proceed to unravel the mystery which has so long surrounded the authorship of Shakespeare's plays, following which instructions he deciphered all that Bacon wished posterity to know.

The story bears upon its face strong evidence in support of the claim that Bacon not only wrote Shakespeare's plays, but many other works. The story of his life and times is scattered all through these works; a line here and a line there; gathered up, according to instructions, and put together, these lines make a most wonderful story indeed. Bacon explains that it was thus hidden for his own safety. He relates that he was the victim of outrageous fortune, cheated out of his rights; and indignant to the last degree, he pronounces "a general curse" on everybody and everything, his enemies in particular. He unfolds a tale that makes one's hair stand on end indeed, "like quills of frightful porcupine." In effect, he clains to be the son of Queen Elizabeth and Robert Dudley, Earl of Leicester, who were secretly married shortly after Elizabeth became queen; that the marriage and his birth were kept secret because of a crime on her part during her girlhood and during the reign of her half-brother, young Edward, by which her life had been forfeited, yet which Edward concealed; that the secret of his birth got out after Bacon became a man, and that Robert Cecil, Lord Burleigh, a dwarf and hunchback—according to the story—having been beaten by young Bacon for calling him a bastard, murdered old Elizabeth to prevent her owning Bacon and announcing him the rightful heir to the throne of England.

While in the main the statements of Bacon comport with history, there is much of it that is either most spicy, highly seasoned unwritten history, or else a court scandal which would not have been at all "to the queen's taste," and which, if false, should have cost him his head, an event, it seems, he greatly feared. It is intensely interesting reading, at all events, and we await parts two and three with impatience. The whole translation is written in the Shakespearean blank verse, and flows as smoothly, and is as full of poetic fancy as any of the alleged works of the "immortal bard." It is out of all reason to suppose that Dr. Owen concocted the story; still more improbable that he should write it in a style so distinctly Shakespearean. But Shakespeare will die hard, if die he must. His friends are legion, and will fight for him to the bitter end. The publishers say:

"The next volume of the deciphered writings of Sir Francis Bacon will be ready for issue the latter part of February. It will contain a continuation of Anne Bacon's account of the circumstances leading up to, and the marriage of Elizabeth to Robert Dudley in the Tower; her accession to the throne; the murder of Amy Robsart Dudley; the banishment of Sir Francis to France, and his account of the Spanish Armada, the most beautiful of all his works.''

Altogether, the publication is the sensation of the century, and Dr. Owen has immortalized himself. The book can be had in paper by enclosing fifty cents to the publishers, at Detroit, or seventy-five cents in cloth binding.

